# Hospital-acquired conditions and length of stay in the pregnancy and puerperal cycle

**DOI:** 10.11606/s1518-8787.2019053000688

**Published:** 2019-08-15

**Authors:** Thales Philipe Rodrigues da Silva, Ariene Silva do Carmo, Taiane Gonçalves Novaes, Larissa Loures Mendes, Alexandra Dias Moreira, Milene Cristine Pessoa, Luna Cosenza, Juliana Fantini Chaves Pereira, Fernanda Penido Matozinhos

**Affiliations:** I Universidade Federal de Minas Gerais. Faculdade de Medicina. Programa de Pós-Graduação em Ciências da Saúde. Belo Horizonte, MG, Brasil; II Universidade Federal de Viçosa. Departamento de Nutrição. Programa de Pós-Graduação em Saúde e Nutrição de Grupos Populacionais. Viçosa, MG, Brasil; III Universidade Federal de Minas Gerais. Escola de Enfermagem. Departamento de Nutrição. Belo Horizonte, MG, Brasil; IV Universidade Federal de Minas Gerais. Escola de Enfermagem. Departamento de Enfermagem Materno-Infantil e Saúde Pública. Belo Horizonte, MG, Brasil; V Instituto de Acreditação e Gestão em Saúde (IAG Saúde). Belo Horizonte, MG, Brasil

**Keywords:** Pregnant Women, Puerperal Disorders, Hospitalization, Length of Stay, economics, Hospital Costs

## Abstract

**OBJECTIVE:**

To analyze the impact of the Hospital-Acquired Conditions (HAC) in women in the puerperal and pregnancy cycle during length of stay.

**METHODS:**

This cross-sectional study was conducted with 113,456 women, between July 2012 and July 2017, in Brazil’s national hospitals of the supplementary healthcare networks and philanthropists accredited to the Unified Health System (SUS). Data on hospital discharges were collected using the Diagnosis-Related Groups (DRG Brasil®) system. All DRGs of the major diagnostic category 14 (MDC14), including pregnancy, childbirth and puerperium, were included. The impact of HAC on length of stay was estimated by Student’s t-test, and the effect size by Cohen’s d, which allows to assess clinical relevance.

**RESULTS:**

The most prevalent diagnostic categories related to MDC14 were vaginal and cesarean deliveries without complicating diagnoses, both at institutions accredited to SUS and those for supplementary health care. The prevalence of HAC was 3.8% in supplementary health and 2.5% in SUS. Hospitals providing services to supplementary health care providers had a longer length of stay considering HAC for patients classified as DRG: cesarean section with complications or comorbidities at admission (p < 0.001; Cohen’s d = 0.74), cesarean section without complications or comorbidities at admission (p < 0.001, Cohen’s d = 0.31), postpartum and post abortion without listed procedure (p < 0.001, Cohen’s d = 1.05), and other antepartum diagnoses with medical complications (p < 0.001; Cohen’s d = 0.77).

**CONCLUSIONS:**

This study showed that the prevalence of HAC was low both in the institutions accredited to attend by SUS and in those of supplementary health; however, its presence contributes to increasing the length of stay in cases of cesarean sections without complications or comorbidities in supplementary health institutions.

## INTRODUCTION

Hospital-acquired conditions ((HAC) are undesirable or adverse events that directly affect the patient’s health and experience in hospital care^1^. They refer to the medical conditions or complications developed during the hospitalization period that were not present at the time of admission^1^. Generally, they result in additional costs, generated both by the increase in hospital stay and by the subsequent treatments^2^. The stay of patients with HAC is, on average, almost four times greater than that of patients without such complications^3^.

Regarding the length of hospitalization, analyses estimate that the average cost of one day of hospitalization in an acute care hospital (with an average hospitalization period of less than 30 days) is € 371^4^. A study that aimed to estimate the daily costs associated with extra hospitalization time to treat HAC, specifically infections, demonstrated that they went from 1.79 to 6.91 days for neurological patients, from 3.76 to 11.3 days for patients attended in the gynecological service and, in the general average, from 0.91 to 8.09 days^5^. We emphasize that length of stay is an indicator of hospital efficiency and is related to the quality of care provided^6^.

Another factor that can contribute to the increase in costs to health, caused by the increase in length of stay, is the adverse event (AE), which may be an HAC^7,8^. The World Health Organization (WHO) defines AE as damage caused by the procedure or complication related to treatment, unrelated to the diagnosis of admission, resulting in prolonged hospitalization or disability present at the time of hospital discharge^8^.

A study conducted in the United States estimated that the total annual cost with AE was U$985 million in 2008 and more than U$ 1 billion in 2009. The average cost per AE for hospitals was U$ 892 in 2008 and increased to U$ 939 in 2009^9^. In Europe, the AEs considered as preventable events represented a total expenditure of € 277,665^10^.

In Brazil, a study that aimed to estimate the financial resources spent on patients with AE in hospitals showed that the mean value per patient with AE considered avoidable (R$ 1,270.47) was 19.5% higher than the mean value per patient without AE. Considering all AEs, the mean value for treating these patients (R$ 3,195.42) was 200.5% higher than that for patients without AE^7^.

The economic impacts of HAC are already well established in the literature^4,5,7,9,10^. However, new studies are necessary to evaluate the economic impact and length of stay in specific populations, especially women in the pregnancy and puerperal cycle. By 2015, about 303,000 women and adolescents died as a result of complications related to pregnancy and childbirth. It is noteworthy that 99% of these maternal deaths occur in contexts of low resources and the majority could be prevented^11^. In Brazil, maternal mortality has declined in recent years, but remains high compared to high-income countries^12^.

The assessment of the impact of HAC on this population may contribute to the improvement of programs that prevent or minimize the occurrence of conditions acquired in the hospital during this period of the reproductive cycle, thus favoring a better quality of care and avoiding potentially fatal complications. Given this problem, the objective of this study was to analyze the impact of the Hospital-Acquired Conditions in women in the pregnancy and puerperal cycle on the length of stay.

## METHODS

This is an epidemiological study with a cross-sectional design, held with 113,456 women between July 2012 and July 2017, in private hospitals that provide services to supplementary health care providers in Brazil and to the Unified Health System (SUS), distributed in all regions of the country and that use Diagnosis-Related Groups (DRG Brasil^®^ version 9). The data were collected from the medical records after discharge and registered in the DRG Brasil^®^ system by nurses dedicated to this function.

DRG or Diagnostic-Related Groups is a methodology for categorizing patients into homogeneous groups according to their characteristics and complexity of treatment. It is applicable to patients admitted to hospitals that attend acute cases, that is, those in which the average hospitalization of the patient does not exceed 30 days^13^. For classification of cases into groups, the following variables are considered: principal diagnosis, patient’s age and sex; comorbidities and complications (secondary diagnoses); and surgical procedures performed^14,15^. For describing the principal, secondary and acquired diagnoses, the International Classification of Diseases (ICD-10) was used. The procedures performed were coded according to the tables used in the SUS and supplementary health (in this case, the Supplementary Healthcare Unified Terminology – TUSS).

Data collection on HAC occurred in two instances: in DRG coders, by reading the medical records and posting the information in DRG Brasil, and by services of care security and hospital infection control, which had been working for years with active search for infectious and non-infectious events in these institutions, in addition to reports of adverse events.

All DRGs that make up the major diagnostic category 14 (MDC14), which includes pregnancy, childbirth and puerperium were included, totaling 15 DRGs (765 to 782). The economic impact was measured indirectly by the variable length of stay, in days.

The sample was described by absolute and relative frequencies. Mean, standard deviation (SD), confidence interval of averages and percentiles of interest (p10, p25, p50, p75 and p90) were presented in relation to the length of stay. The results were presented considering the DRG and the paying source (supplementary health or SUS). In addition, statistics were calculated for the overall length of hospital stay, considering whether acquired conditions occurred or not.

The comparative analysis between two groups of patients (with and without HAC) regarding the stay for each DRG was performed using Student’s t-test for independent samples. In cases where the analysis indicated a significant difference (p < 0.05), the size of this effect was evaluated. Because these are large samples, there is an increase in the probability of type I error; therefore, effect size measurement by Cohen’s D allows the evaluation of clinical relevance. Thus, only factors with a significant effect and effect size equal to or greater than 0.30 were considered. Data were processed and analyzed using the free software R.

The project was approved by the Research Ethics Committee, number 34133814.5.0000.5149. Exemption from the free and informed consent form was obtained.

## RESULTS

Among the diagnostic categories related to pregnancy, childbirth and puerperium, vaginal delivery without complicating diagnoses (DRG 775) was the most prevalent in public care (45.8%). On the other hand, in supplementary health, cesarean section without complications or comorbidities at admission (DRG 766) was the most prevalent (49.6%) (Table 1).

**Table 1:** Characterization of payment source and most frequent DRGs of the diagnostic categories related to pregnancy, childbirth and puerperium. Brazil, 2012–2017.

Diagnostic categories		Payment source			
	
DRG	Description	Public service		Supplementary health	
	
		n	%	n	%
765	Cesarean section with CC/MCC	1,036	7.83	12,252	12.22
766	Cesarean section without CC/MCC	3,990	30.17	49,707	49.59
767	Vaginal delivery with sterilization and/or dilation and curettage	70	0.53	419	0.42
768	Vaginal delivery with O.R. procedure except sterilization and/or dilatation and curettage	2	0.02	17	0.02
769	Postpartum and post abortion diagnoses with O.R. procedure	146	1.10	432	0.43
770	Abortion with dilation and curettage, aspiration curettage or hysterectomy	743	5.62	8,331	8.31
774	Vaginal delivery with complicating diagnoses	204	1.54	858	0.86
775	Vaginal delivery without complicating diagnoses	6,055	45.78	21,694	21.64
776	Postpartum and post abortion diagnoses without listed procedure	97	0.73	671	0.67
777	Ectopic pregnancy	88	0.67	1,100	1.10
778	Threatened abortion	123	0.93	1,033	1.03
779	Abortion without dilatation and curettage	21	0.16	323	0.32
780	False labor	33	1.25	199	0.20
781	Other antepartum diagnoses with medical complications	317	2.40	2,135	2.13
782	Other antepartum diagnoses without medical complications	302	2.28	1,058	1.06
Total		13,227		100,229	

DRG: Diagnosis-Related Groups; CC: complications or comorbidities at admission; MCC: major complications or comorbidities (very significant), additional to initial diagnosis

Cesarean sections with and without complications at admission represented more than half (61.8%) of all prevalent hospitalizations / in supplementary health, being present in 38.0% of cases in public care. In relation to vaginal deliveries with or without complications, the prevalence of hospitalization was 47.9% in public care and 22.9% in supplementary health (Table 1).

HAC occurred in 3.8% of hospitalizations in supplementary health and 2.5% in public care. Among HAC by women in the pregnancy and puerperal cycle, the most prevalent in supplementary healthcare services were: second degree perineal laceration during delivery (20.3%), infection of obstetric surgical wound (9.4%), delayed and secondary postpartum hemorrhage (8.2%), and spinal and epidural anesthesia-induced headache during the puerperium (7.4%). In public service, the most frequent acquired conditions were perineal lacerations during delivery, with those of second degree corresponding to 73.4% of the cases – 5.7% were first degree and 4.2% third degree (data not shown).

Regarding the overall length of stay and length of hospitalization, excluding the HAC, the cesarean section with complications or comorbidities at admission (DRG 765) was responsible for the highest rates for public care, with a mean of 3.8 days (CI95% 3.69–3.91) in both indicators (Table 2). In supplementary health, cesarean section with complications or comorbidities at admission and postpartum and post abortion diagnoses without O.R. procedure (DRG 776) were responsible for a longer hospitalization (Table 3).

**Table 2:** Characterization of hospitalizations in public service in relation to the stay according to the diagnostic category related to pregnancy, childbirth and puerperium. Brazil, 2012–2017.

Public service								

DRG	n	x̅ (DP)	CI95% (x̅ )	p10	p25	p50	p75	p90
	Overall length of stay (days)							

765	1,036	3.8 (1.8)	3.69–3.91	2.1	2.6	3.1	4.8	9.0
766	3,990	2.8 (1.4)	2.76–2.84	2.1	2.2	2.8	3.1	4.0
767	70	2.2 (1.7)	1.80–2.60	1.2	1.5	2.1	3.0	4.5
769	146	3.1 (1.8)	2.81–3.39	1.7	3.0	3.0	3.0	6.3
770	743	1.1 (1.7)	0.98–1.22	0.6	0.8	1.0	1.5	2.0
774	204	2.8 (1.7)	2.57–3.03	1.6	1.9	2.7	3.6	5.6
775	6,055	2.0 (1.4)	1.96–2.04	1.3	1.5	1.9	2.4	3.0
776	97	2.9 (2.2)	2.46–3.34	1.1	1.7	2.7	5.0	7.9
777	88	2.5 (1.6)	2.17–2.83	1.7	1.9	2.2	2.9	4.5
778	123	2.0 (2.5)	1.56–2.44	0.6	1.0	2.5	3.7	5.0
780	33	1.5 (2.3)	0.72–2.28	0.7	0.9	1.4	2.5	4.1
781	317	3.0 (2.1)	2.77–3.23	1.2	1.9	2.8	4.7	7.8
782	302	2.9 (2.2)	2.65–3.15	1.1	1.8	3.0	4.6	7.8

	Length of stay excluding Hospital- Acquired Conditions - (days)							

765	1,018	3.8 (1.8)	3.69–3.91	2.1	2.6	3.0	4.7	8.8
766	3,980	2.8 (1.4)	2.76–2.84	2.1	2.2	2.8	3.1	4.0
767	59	2.3 (1.8)	1.84–2.76	1.2	1.5	2.2	3.0	5.3
769	145	3.1 (1.7)	2.82–3.38	1.7	3.0	3.0	3.0	5.5
770	742	1.1 (1.7)	0.98–1.22	0.6	0.8	1.0	1.5	2.0
774	186	2.8 (1.7)	2.56–3.04	1.6	1.9	2.7	3.6	5.2
775	5,791	2.0 (1.5)	1.96–2.04	1.3	1.5	1.9	2.4	3.0
776	94	2.9 (2.2)	2.46–3.34	1.0	1.7	2.7	5.0	7.9
777	88	2.5 (1.6)	2.17–2.83	1.7	1.9	2.2	2.9	4.5
778	123	2.0 (2.5)	1.56–2.44	0.6	1.0	2.5	3.7	5.0
780	33	1.5 (2.3)	0.72–2.28	0.7	0.9	1.4	2.5	4.1
781	315	2.9 (2.1)	2.67–3.13	1.2	1.9	2.8	4.7	7.8
782	299	2.9 (2.2)	2.65–3.15	1.1	1.8	3.0	4.6	7.8

DRG: Diagnosis-Related Groups

**Table 3:** Characterization of hospitalizations of supplementary health in relation to stay due to diagnostic category related to pregnancy, childbirth and puerperium. Brazil, 2012–2017.

Supplementary health								

DRG	n	x̅ (dp)	CI95% (x̅ )	p10	p25	p50	p75	p90
	Overall length of stay (days)							

765	12,252	2.6 (1.7)	2.57–2.63	1.7	2.0	2.2	3.0	5.2
766	49,707	2.2 (1.3)	2.19–2.21	1.7	1.9	2.1	2.3	2.9
767	419	2.0 (1.9)	1.82–2.18	1.1	1.4	1.9	2.6	4.5
769	432	1.5 (3.5)	1.17–1.83	0.3	0.7	1.6	3.2	7.7
770	8,331	0.7 (2.1)	0.65–0.75	0.3	0.4	0.7	1.0	1.5
774	858	2.3 (1.8)	2.18–2.42	1.3	1.6	2.1	2.7	4.1
775	21,694	1.8 (1.5)	1.78–1.82	1.2	1.4	1.8	2.2	2.7
776	671	2.7 (2.4)	2.52–2.88	0.8	1.7	2.9	4.7	7.1
777	1,100	1.6 (1.7)	1.50–1.70	0.8	1.1	1.7	2.0	2.8
778	1,033	1.9 (2.4)	1.75–2.05	0.6	1.1	1.8	3.0	5.5
779	323	1.1 (2.4)	0.84–1.36	0.4	0.7	1.1	1.9	3.0
780	199	1.3 (2.6)	0.94–1.66	0.4	0.7	1.6	2.4	3.9
781	2,135	2.2 (2.2)	2.11–2.29	0.8	1.4	2.3	3.7	5.7
782	1,058	2.3 (2.4)	2.16–2.44	0.8	1.4	2.2	3.9	6.9

	Length of stay excluding Hospital- Acquired Conditions (days)							

765	11,453	2.6 (1.6)	2.57–2.63	1.7	2.0	2.2	2.9	4.8
766	48,581	2.1 (1.3)	2.09–2.11	1.7	1.9	2.1	2.3	2.8
767	331	2.0 (1.9)	1.80–2.20	1.0	1.4	1.9	2.6	4.2
769	404	1.4 (3.3)	1.08–1.72	0.3	0.7	1.5	3.0	6.3
770	8,273	0.7 (2.1)	0.65–0.75	0.3	0.4	0.7	1.0	1.5
774	777	2.2 (1.8)	2.07–2.33	1.3	1.6	2.1	2.7	4.1
775	20,214	1.8 (1.5)	1.78–1.82	1.2	1.4	1.7	2.2	2.7
776	639	2.6 (2.4)	2.41–2.79	0.8	1.7	2.9	4.7	6.8
777	1,079	1.6 (1.7)	1.50–1.70	0.8	1.1	1.7	2.0	2.8
778	1,016	1.8 (2.4)	1.65–1.95	0.6	1.1	1.8	2.9	5.3
779	322	1.1 (2.4)	0.84–1.36	0.4	0.7	1.1	1.8	3.0
780	198	1.3 (2.6)	0.94–1.66	0.4	0.7	1.6	2.4	3.9
781	2,072	2.2 (2.2)	2.11–2.29	0.8	1.4	2.2	3.6	5.6
782	1,050	2.3 (2.4)	2.15–2.45	0.8	1.4	2.2	3.9	6.9

DRG: Diagnosis-Related Groups

Table 4 presents a comparative analysis between groups of patients from supplementary health with and without acquired conditions. We observed a longer hospital stay in the presence of AHC for patients categorized in DRG: cesarean section with complications or comorbidities at admission (DRG 765); cesarean section without complications or comorbidities at admission (DRG 766); vaginal delivery with sterilization and/or dilatation and curettage (DRG 767); abortion with dilation and curettage, aspiration curettage or hysterectomy (DRG 770); vaginal delivery without complicating diagnoses (DRG 775); postpartum and post abortion diseases without O.R. procedure (DRG 776); and other antepartum diagnoses with medical complications (DRG 781). When analyzing Cohen’s d values, the HAC were related to a longer hospital stay in DRG 765 (3.8 days *versus* 2.6 days, p < 0.001, Cohen’s d = 0.74), DRG 766 (2.5 days *versus* 2.1 days, p < 0.001, Cohen’s d = 0.31), DRG 776 (5.1 days *versus* 2.6 days, p < 0.001, Cohen’s d = 1.05), and DRG 781 (3.9 days *versus* 2.2 days, p < 0.001, Cohen’s d = 0.77).

**Table 4:** Comparative analysis between group of patients with Hospital- Acquired Conditions and group of patients without acquired conditions in relation to the stay (in days), in supplementary health. Brazil, 2012–2017.

DRG	With Hospital- Acquired Conditions			Without Hospital- Acquired Conditions			p	Cohen’s d
	
	n	x̅ (dp)	95%CI (x̅ )	n	x̅ (dp)	95%CI (x̅ )		
765	799	3.8 (2.0)	3.64–3.93	11,453	2.6 (1.6)	2.57–2.63	**< 0.001**	**0.74**
766	1,126	2.5 (1.5)	2.45–2.63	48,581	2.1 (1.3)	2.09–2.11	**< 0.001**	**0.31**
767	88	2.3 (1.8)	1.96–2.71	331	2.0 (1.9)	1.80–2.20	**0.018**	0.16
770	57	1.2 (2.9)	0.45–1.97	8,273	0.7 (2.1)	0.65–0.75	**0.001**	0.24
774	81	2.5 (1.8)	2.07–2.83	777	2.2 (1.8)	2.07–2.33	0.142	0.17
775	1,480	1.9 (1.5)	1.83–1.98	20,214	1.8 (1.5)	1.78–1.82	**< 0.001**	0.07
776	32	5.1 (2.1)	4.42–5.84	639	2.6 (2.4)	2.41–2.79	**< 0.001**	**1.05**
781	63	3.9 (2.0)	3.44–4.42	2,072	2.2 (2.2)	2.11–2.29	**< 0.001**	**0.77**

DRG: Diagnosis-Related Groups

p-value in bold < 0.05 according to Student’s t-test for independent samples.

We emphasize that, for the other supplementary health categories, as well as for all categories of public care, no differences were found between the overall length of stay and length of hospitalization, excluding the acquired conditions.

## DISCUSSION

This study showed that the most prevalent diagnostic categories related to pregnancy, childbirth and puerperium were vaginal deliveries without complicating diagnoses and cesarean sections, both in public and supplementary health care institutions. The HAC increased the length of stay in cases of cesarean section with complications or comorbidities at admission, cesarean section without complications or comorbidities at admission, pospartum and post abortion diseases without O.R. procedure, and other antepartum diagnoses with medical complications in the hospitals that provide services to supplementary health care providers in Brazil.

Cesarean sections with complications or comorbidities at admission are among the DRGs that most contributed to the longest period of hospitalization, with an average of 3.8 and 2.6 days in the public and private sectors, respectively. Studies show that the length of hospital stay for cesarean sections is higher than for vaginal delivery^16^. One of the probable reasons for this difference is the slow wound healing process and the long period of convalescence in cesarean sections^16^. The prolonged hospitalization time of cesarean sections is one of the factors that contributes to the higher hospital cost of this procedure^14^.

According to the WHO, cesarean rates above 10% are not associated with the reduction of maternal and neonatal mortality, and this practice should be performed only when necessary^15^. The rates of cesarean sections found in this study, both in supplementary health (72.94%) and in public care (44.25%), are higher than those recommended by the WHO.

Rates of cesarean deliveries increased considerably in several countries^17^. In Brazil, it was 15% in the 1970s, 30% in the early 1980s, reached 40% in the early 1990s, and stabilized in the 2000s^18^. According to data from the study “Birth in Brazil: national enquiry into labor and birth” from 2011–2012, the cesarean rate in the private sector is higher than that found in the public sector (87.9% *versus* 42.9%, respectively)^19^. There are several factors that favor the increase of cesarean sections, especially in the private sector, such as: financial reimbursement offered by Brazilian supplementary health insurance, infrastructure issues, qualification human resources, cultural factors and maternal request^20,21^.

We can infer that in the private sector, cesarean sections are not predominantly related to the presence of obstetric risk, since rates are high in low-risk women^19^. In addition, around 84.2% of all cesarean sections in Brazil are performed before the active stage of labor^19^. This scenario may contribute to increased maternal and neonatal morbidity and mortality, especially when surgery is performed before 39 weeks of gestational age^22^.

Given this context, in 2016, the Federal Medical Council (CFM) established criteria for cesarean section at the request of women in Brazil. It established that, in situations of habitual risk, it could be performed only from the 39th week of gestation^23^. In addition, the National Regulatory Agency for Private Health Insurance and Plans (ANS) implemented some measures to encourage normal childbirth, such as mandatory use of the partograph and the pregnant woman’s card. The same resolution determines the right of access of the population to the percentage of cesarean sections performed by a health plan, establishment and physician^24^. However, it is crucial to think of other institutional and organizational strategies of the health care networks, seeking changes in the paradigm of obstetric care, in order to conduct the birth process more physiologically^25,26^ and, consequently, reduce unnecessary hospital expenses and conditions acquired in the pregnancy and puerperal cycle.

We emphasize that in our study, the HAC with the cesarean section without complication or comorbidity increased the length of hospital stay in the supplementary health sector, being more frequent conditions the infections of surgical wounds, hemorrhages in the immediate postpartum, lacerations and headache related to anesthetic procedure. Besides increasing hospital costs, elective cesarean sections are associated with an increased risk of maternal mortality and severe obstetric complications^27^.

A recent meta-analysis evaluated acute maternal complications related to cesarean sections without indication. Women who underwent cesarean deliveries had nearly a threefold increase in the chance of infection compared to those undergoing vaginal delivery, and a greater chance of being admitted to an intensive care unit. On the other hand, vaginal deliveries have a greater chance of obstetric trauma and bleeding, however with degree of weak evidence^28^.

Postpartum hemorrhage is the main cause of maternal mortality in the world^29^, besides predicting other complications, such as acute renal failure and disseminated intravascular coagulation. Health professionals should therefore be familiar with identifying the causes of hemorrhage (uterine atony, lacerations, retained placenta) and their respective procedures, such as administration of uterotonics^30^.

The coincidence between length of stay and length of hospitalization excluding the HAC in the other DRGs may be due to the low prevalence (less than 5%) of adverse conditions observed among the women evaluated. Another finding of this study is that the impact of a longer stay on women in the pregnancy and puerperal cycle is associated with higher costs for services. The reduction of hospital costs has become a constant concern among health administrators^31^. Hospital institutions began to pay attention to this aspect, but trying to maintain an excellent service and ensure customer satisfaction; to that end, patient safety was established as the main objective^32^. Therefore, the literature emphasizes that HAC may be a result of problems in practice, products, processes or systems, and that their occurrence results from a chain of systemic factors^1^. We reinforce that studies in hospitals in several countries show the association between HAC and increased length of stay, one of the patient safety indicators^3,6,33,34^.

As limitations, the study presents low sample representativeness of the public sector, making impossible the statistic comparison of confidence intervals of the averages of public health care and those of supplementary health. Another limitation is the lack of information on the remuneration mechanism, which is known to influence the length of stay of women and the coding quality of the HAC. We should also highlight the potential issues of the study, such as the use of data from the DRG system, which presents good representation for supplementary health.

This study showed low prevalence of HAC, contributing to the increase of length of stay in cases of cesarean sections without complications or comorbidities in supplementary health. These data suggest the need for strategies that recommend surgical procedure through precise indications, considering clinical and obstetric criteria, which may contribute to greater safety and protection of maternal and neonatal health, as well as the optimization of hospital expenses.
